# Risk of cardiovascular disease in patients with fatty liver disease as defined from the metabolic dysfunction associated fatty liver disease or nonalcoholic fatty liver disease point of view: a retrospective nationwide claims database study in Japan

**DOI:** 10.1007/s00535-021-01828-6

**Published:** 2021-10-03

**Authors:** Masato Yoneda, Takuma Yamamoto, Yasushi Honda, Kento Imajo, Yuji Ogawa, Takaomi Kessoku, Takashi Kobayashi, Asako Nogami, Takuma Higurashi, Shingo Kato, Kunihiro Hosono, Satoru Saito, Atsushi Nakajima

**Affiliations:** 1 Department of Gastroenterology and Hepatology, Yokohama City University Hospital, 3-9 Fukuura, Kanazawaku, Yokohama 236-0004 Japan; 2 Cardiovascular and Diabetes, Product Marketing Department, Kowa Company, Ltd., 3-4-10 Nihonbashi Honcho Tokyo, 103-0023, Japan; 3 Department of Gastroenterology, Shin-Yurigaoka General Hospital, 255 Furusawatsuko, Asao, Kawasaki 215-0026 Japan; 4 Department of Gastroenterology, National Hospital Organization Yokohama Medical Center, 3-60-2 Harajyuku, Totsuka, Yokohama 245-8675 Japan

**Keywords:** Cardiovascular disease, Diabetes mellitus, Hypertriglyceridemia, Non-alcoholic fatty liver disease, Metabolic dysfunction associated fatty liver disease

## Abstract

**Background:**

Nonalcoholic fatty liver disease (NAFLD) and metabolic dysfunction associated fatty liver disease (MAFLD) have important associations with cardiovascular disease (CVD). The main objective of this study was to compare the frequency of incidence rate of CVD in the NAFLD or MAFLD patients utilizing a large claims database.

**Methods:**

Using the JMDC database from April 2013 to March 2019, we retrospectively analyzed data for 1,542,688 and 2,452,949 people to estimate the relationship between CVD and NAFLD, MAFLD, respectively.

**Results:**

The incidence rates of CVD were 0.97 (95% CI 0.94–1.01) and 2.82 (95% CI 2.64–3.01) per 1000 person-years in the non-NAFLD and NAFLD groups, respectively, and 1.01 (95% CI 0.98–1.03) and 2.69 (95% CI 2.55–2.83) per 1000 person-years in the non-MAFLD and MAFLD groups, respectively. The overall prevalence of hypertriglyceridemia and diabetes mellitus (DM) was 13.1, and 4.2%, respectively, in the non-NAFLD group and 63.6, and 20.2%, respectively, in the NAFLD group. The overall prevalenceof hypertriglyceridemia and DM was 13.6 and 4.3%, respectively, in the non-MAFLD group and 64.1, and 20.6%, respectively, in the MAFLD group. HRs for CVD increased with hypertriglyceridemia and DM.

**Conclusions:**

Results indicated that incident rate of CVD increased with NAFLD/MAFLD; the complication rate of DM and hypertriglyceridemia among NAFLD/MAFLD patients is high and may affect the development of CVD.

**Supplementary Information:**

The online version contains supplementary material available at 10.1007/s00535-021-01828-6.

## Introduction

Non-alcoholic fatty liver disease (NAFLD) is the most common liver disease worldwide with a global prevalence of 25.2% and a prevalence of 29.6% in Asia [1, 2]. NAFLD is regarded as a hepatic component of metabolic syndrome and is associated with other risk factors for metabolic syndrome, such as obesity, diabetes mellitus (DM), and dyslipidemia [1, 3]. Recently, fatty liver caused by nutritional metabolic disorders regardless of other chronic liver diseases has been proposed as a new liver disease concept, “metabolic dysfunction associated fatty liver disease (MAFLD)" [4]. Cardiovascular disease (CVD) has been reported as the most important cause of death, followed by non-liver malignancy and complications of cirrhosis (along with hepatocellular carcinoma and liver transplantation) in NAFLD patients [5, 6]. Furthermore, the accumulation of fat in the liver is reported to be independently associated with coronary plaques, especially non-calcified plaques [7], and both hepatic steatosis and fibrosis are significantly associated with diastolic heart dysfunction [8].

Multiple reports have indicated that NAFLD might have had important associations with cardiovascular outcomes in the past decade [9, 10]. Some reports have shown that MAFLD correlates more strongly with CVD than NAFLD [11, 12]. However, the link between NAFLD/MAFLD and CVD is more complex than previously thought, and it remains unclear how NAFLD/MAFLD is associated with the development of CVD [13].

Recently, a cohort study that enrolled 120,795 NAFLD patients with matched controls extracted from primary healthcare databases from four European countries reported no association between NAFLD and the risk of acute myocardial infarction (AMI) or stroke, after adjustments for established cardiovascular risk factors [14]. CVD may affect various populations differently. The incidence of organic coronary artery disease (CAD), a major cause of heart failure in Western countries, is relatively low in East Asian countries [15]. The age-adjusted death rate resulting from ischemic heart disease in Japan was estimated to be one-sixth of that in the United States [16].

Therefore, we undertook a longitudinal analysis of NAFLD/MAFLD based on prescription records derived from a large nationwide administrative claims database to estimate the incident risk of developing CVD in cohorts encountered in routine practice. The main objective of this study was to compare the incidence rate of CVD in the NAFLD and non-NAFLD groups. Furthermore, the same study was also conducted in the MAFLD group and the non-MAFLD group.

## Methods

### Large claims database

A large claims database constructed by the Japan Medical Data Center (JMDC Co., Ltd. Tokyo, Japan), using standardized disease classifications and anonymous record linkage [17], was used in this retrospective cohort study. This claims database contains monthly claims from medical institutions and pharmacies submitted from January 2005 to April 2020 and includes records of approximately 9.6 million insured persons, comprising mainly company employees and their family members.

This study analyzed 6.8 million persons who were registered in this database from April 2013 to March 2019. The JMDC database provides information on the beneficiaries, including encrypted personal identifiers, age, sex, data from health check-ups, questionnaire information from several insurance unions or International Classification of Diseases 10th revision (ICD-10) codes from the Ministry of Health, Labor and Welfare of Japan, diagnostic codes, and treatment fee codes. In addition, information concerning the name, dose, and number of days a prescribed drug was supplied and/or dispensed was obtained from the database. All medications were coded according to the Anatomical Therapeutic Chemical Classification of the European Pharmaceutical Market Research Association. An encrypted personal identifier was used to link claims data from different hospitals, clinics, and pharmacies. Based on the JMDC database, we defined our study cohort to enable us focus on CVD onset. The Ethics committee/institutional review board of Yokohama City University determined that ethics approval was not required because personal information in the claims database used in this study was completely deleted.

### NAFLD patients and study design

**Fig. 1:**
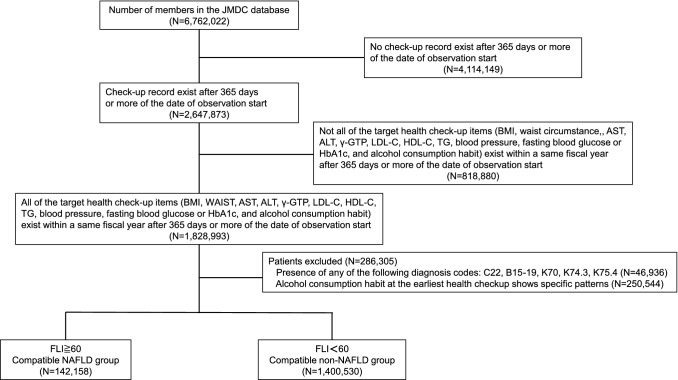
Flowchart of the NAFLD subjects enrollment

Figure 1 shows the flowchart for patient selection (NAFLD subjects). People who underwent a medical check-up between April 2013 and March 2019 were selected from the JMDC claims database (*n* = 6,762,022). People for whom all health check-up data [body mass index (BMI), waist circumstance, sex, age, aspartate transaminase (AST), alanine aminotransferase (ALT), γ-glutamyl transpeptidase (GGT), low-density lipoprotein cholesterol (LDL-C), high-density lipoprotein cholesterol (HDL-C), triglyceride (TG), blood pressure, fasting blood glucose or HbA1c, and questionnaire on alcohol habits) were available within the same year and ≥ 365 days after the date of the initial observation were enrolled (*n* = 1,828,993). Among the patients, those with malignant neoplasms of the liver and/or intrahepatic bile ducts [ICD-10 code (ICD10): C22], viral hepatitis (ICD10: B15-19), alcoholic liver disease (ICD10: K70), primary biliary cirrhosis (ICD10: K74.3), autoimmune hepatitis (ICD10: K75.4), and excessive alcohol drinking habit (Supplementary Table 1) were excluded. Finally, 1,542,688 patients were included in this analysis.

Because the JMDC database does not contain information such as liver imaging or histology results, a definitive diagnosis of NAFLD could not be made based on database records alone. Thus, NAFLD patients were defined as patients with (1) no secondary cause of liver injury such as significant alcohol consumption, viral hepatitis, autoimmune hepatitis, primary biliary cholangitis (or primary biliary cirrhosis), or malignant neoplasm of the liver and/or intrahepatic bile ducts and (2) the presence of fatty liver was defined using the Fatty Liver Index (FLI) prediction model [18–21]. FLI was calculated using the following formula: ${\text{FLI}} = \left( {{\text{e}} ^{{0.{953}*{\text{loge }}\left( {{\text{triglycerides}}} \right) + 0.{139}*{\text{BMI}} + 0.{718}*{\text{loge }}\left( {{\text{ggt}}} \right) + 0.0{53}*{\text{waist circumference}} - {15}.{745}}} } \right)/\left( {{1} + {\text{e}} ^{{0.{953}*{\text{loge }}\left( {{\text{triglycerides}}} \right) + 0.{139}*{\text{BMI}} + 0.{718}*{\text{loge }}\left( {{\text{ggt}}} \right) + 0.0{53}*{\text{waist circumference}} - {15}.{745}}} } \right) \, \times { 1}00$ [18]. In this study, the presence of steatosis was diagnosed based on a FLI ≥ 60 that identified hepatic steatosis [21].

### MAFLD patients and study design

**Fig. 2:**
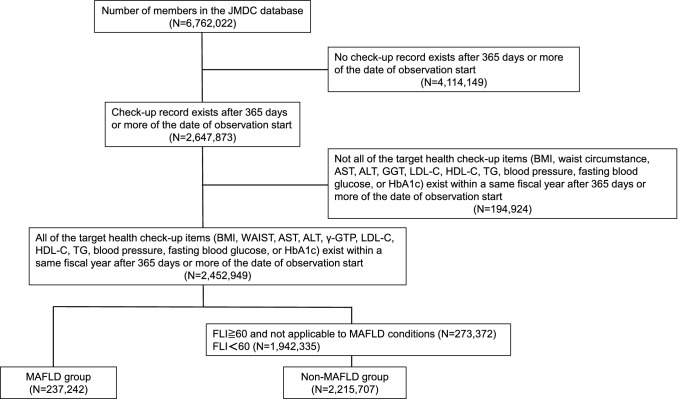
Flowchart of the MAFLD subjects enrollment

Figure 2 shows the flowchart for patient selection of the MAFLD cohort. Patients who underwent a medical check-up between April 2013 and March 2019 were selected from the JMDC claims database (*n* = 6,762,022). Patients for whom all health check-up data (BMI, waist circumstance, AST, ALT, GGT, LDL-C, HDL-C, TG, blood pressure, fasting blood glucose, or HbA1c) were available within the same year and ≥ 365 days after the date of the initial observation were enrolled (*n* = 2,452,499). In this study, MAFLD was diagnosed based on a FLI ≥ 60 that identified hepatic steatosis [21], associated with the presence of any one of the following three metabolic conditions: diabetes mellitus, overweight/obesity, or metabolic syndrome [4]. According to the definition of MAFLD [4], overweight patients of Asian origin were defined as those with a body mass index (BMI) ≥ 23 kg/m^2^. Metabolic dysregulation was defined as the presence of two or more of the following conditions: (1) waist circumference ≥ 102 in men and 88 cm in women, (2) blood pressure ≥ 130/85 mmHg or specific drug treatment, (3) TG ≥ 150 mg/dl or specific drug treatment, (4) HDL-C < 40 mg/dl for men and < 50 mg/dl for women or specific drug treatment, (5) prediabetes (i.e., fasting glucose levels 100–125 mg/dl, or 2-h post-load glucose levels 140 to 199 mg/dl or HbA1c 5.7–6.4%, (6) Homeostasis model assessment of insulin resistance score ≥ 2.5, and (7) Plasma high-sensitivity C-reactive protein level > 2 mg/L [4]. Since the JMDC database does not have data on 2-h post load glucose levels, HOMA-R, and high-sensitivity C-reactive protein, these factors were not used.

### Hypertriglyceridemia, hypertension, and diabetes mellitus

Hypertriglyceridemia was defined based on a prescription of anti-hypertriglyceridemia medication or a serum TG level ≥ 150 mg/dL with no anti-triglyceride medication. Hypertension was defined if the patient was receiving antihypertensive medication and ICD-10 code for hypertension (ICD10: I10), or had a systolic blood pressure ≥ 140 mm Hg or diastolic blood pressure ≥ 90 mm Hg. DM was defined based on a prescription of antidiabetic medication or a fasting blood glucose level ≥ 126 mg/dL or a HbA1c level ≥ 6.5% with no antidiabetic medication [22] (Supplementary Table 2).

### Study outcome and definitions

The primary study outcome was the incidence rate of CVD, which was defined as procedure code or the combination of ICD codes and procedure code (Supplementary Table 2). The treatment fee codes for percutaneous coronary intervention are K546, K547, K548, and K549. The treatment fee code for coronary artery bypass grafting is K552. The ICD-10 code for cerebral infarction is I63, and the treatment fee codes for cerebral infarction are K149, K164, K178, K609, and K610. CVD (cerebral infarction + coronary artery event) were measured during the follow-up period after the initiation of study recruitment in NAFLD and MAFLD patients, and compared with non-NAFLD and non-MAFLD patients, respectively. The secondary outcome was the hazard ratio of CVD with or without hypertriglyceridemia and/or DM in NAFLD and non-NAFLD patients.

### Patient and public involvement

No patients were involved in the setting of this study or the analysis of the results. However, the Japanese Society of Gastroenterology has published guidelines for the public on the Internet, and the public is also becoming more interested in the relationship between NAFLD and cardiovascular disease [23].

### Statistical analysis

Categorical variables are summarized as percentages, and continuous variables are summarized as means ± standard deviations. Between-group comparisons were evaluated using the Student’s *t* test or Chi-square test, as appropriate. Within the NAFLD and non-NAFLD groups, and MAFLD and non-MAFLD groups, we estimated the incidence rates of coronary artery events, cerebral infarctions, and CVD by dividing the number of incident events by the total number of person years at risk. The corresponding 95% confidence intervals were estimated assuming a Poisson distribution. The hazard ratios for incident coronary artery events, cerebral infarction, and CVD associated with a diagnosis of NAFLD were independently estimated using the Cox proportional hazards models. The models were adjusted by (1) age, sex, and smoking habit and (2) age, sex, smoking habit, body mass index (BMI), LDL-C, hypertension, DM, hypertriglyceridemia, and statin use in multivariable models. The hazard ratios for ischemic coronary events, cerebral infarction, and CVD events associated with a diagnosis of MAFLD were independently estimated using the Cox proportional hazards models. The models were adjusted by (1) age, sex, and smoking habit and (2) age, sex, smoking habit, LDL-C, and statin use in multivariable models. Hazard ratios for coronary artery events, cerebral infarction, and CVD are measured with or without hypertriglyceridemia and/or DM after adjusted by age, sex, smoking habit, BMI, LDL-C, hypertension, and statin use in NAFLD compared with non-NAFLD groups. Directed acyclic graph (DAG) was presented to explain our theory (supplementary Fig. 1) [24].

## Results

### Cohort characteristics

**Fig. 3:**
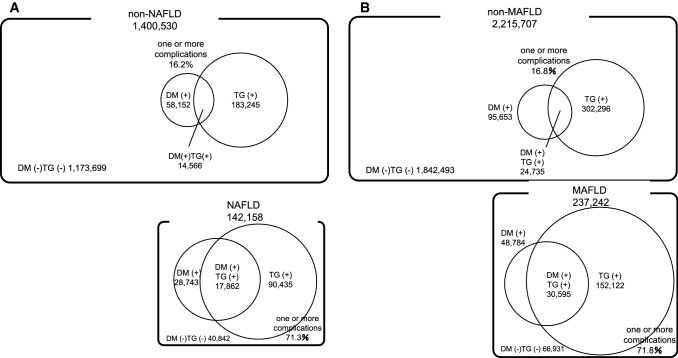
Prevalence of hypertriglyceridemia and diabetes (**A**) in non-NAFLD and NAFLD patients, (**B**) in non-MAFLD and MAFLD patients. *NAFLD* non-alcoholic fatty liver disease, *MAFLD* metabolic dysfunction associated fatty liver disease

In total, 1,542,688 patients were included in the NAFLD cohort, while 2,452,949 patients were included in the MAFLD cohort. The prevalence of NAFLD was estimated to be 9.2% (*n* = 142,158), while MAFLD was estimated to be 9.7% (*n* = 237,242). The demographic and clinical characteristics of the NAFLD and non-NAFLD groups, and MAFLD and non-MAFLD groups are shown in Table 1. The prescription history of each group is shown in Supplemental Tables 3 and 4. Traditional cardiovascular risk factors were more common in the NAFLD and MAFLD groups than in the non-NAFLD and non-MAFLD groups. Specifically, the percentage of smokers and the male-to-female ratio were much higher in the NAFLD and MAFLD groups than in the non-NAFLD and non-MAFLD groups. Furthermore, the serum LDL-C level, serum TG level, systolic blood pressure, diastolic blood pressure, fasting blood glucose, and HbA1c level were significantly higher and the serum HDL-C level was significantly lower in the NAFLD and MAFLD groups than in the non-NAFLD and non-MAFLD groups. The overall prevalence of hypertriglyceridemia, DM, and the combination of hypertriglyceridemia and DM were 13.1%, 4.2%, and 1.0%, respectively, in the non-NAFLD group and 63.6%, 20.2%, and 12.6%, respectively, in the NAFLD group (Table 1, Fig. 3a). The overall prevalence of hypertriglyceridemia, DM, and the combination of hypertriglyceridemia and DM were 13.6%, 4.3%, and 1.1%, respectively, in the non-MAFLD group and 64.1%, 20.6%, and 12.9%, respectively, in the MAFLD group (Table 1, Fig. 3b).

**Table 1:** Clinical characteristics of study participants in the NAFLD cohort and MAFLD cohort

	non-NAFLD (*n* = 1,400,530)	NAFLD (*n* = 142,158)	*p* value		non-MAFLD (*n* = 2,215,707)	MAFLD (*n* = 237,242)	*p* value
Median (interquartile range) follow up (y)	4.00 (2.93–5.82)	3.99 (2.92–5.88)	0.26		3.99 (2.89–5.81)	3.99 (2.90–5.86)	0.33
Age (y)	46.0 ± 10.5	46.2 ± 9.3	< .0001		45.8 ± 10.7	46.0 ± 9.5	< .0001
BMI (kg/m^2^)	22.3 ± 2.9	30.0 ± 3.7	< .0001		22.3 ± 2.9	30.0 ± 3.8	< .0001
Male *n*, (%)	851,850 (60.8)	121,301 (85.3)	< .0001		1,319,577 (59.6)	200,455 (84.5)	< .0001
LDL-C (mg/dL)	120.2 ± 30.4	133.6 ± 33.0	< .0001		119.3 ± 30.7	132.5 ± 33.4	< .0001
HDL-C (mg/dL)	64.3 ± 16.1	48.0 ± 10.4	< .0001		64.7 ± 16.4	48.5 ± 10.8	< .0001
TG (mg/dL)	94.4 ± 57.9	219.6 ± 162.7	< .0001		95.8 ± 61.1	225.2 ± 171.7	< .0001
SBP (mmHg)	118.2 ± 15.7	130.8 ± 15.4	< .0001		118.7 ± 15.8	131.2 ± 15.5	< .0001
DBP (mmHg)	73.0 ± 11.4	82.4 ± 11.3	< .0001		73.2 ± 11.5	82.5 ± 11.3	< .0001
AST (U/L)	20.9 ± 7.4	33.1 ± 17.7	< .0001		21.2 ± 8.5	34.1 ± 20.4	< .0001
ALT (U/L)	20.3 ± 11.9	51.7 ± 33.0	< .0001		20.5 ± 12.6	52.2 ± 34.8	< .0001
GGT (U/L)	31.5 ± 31.8	68.1 ± 60.4	< .0001		33.5 ± 38.9	74.0 ± 73.1	< .0001
FBG (mg/dL)	93.7 ± 15.0	107.1 ± 28.8	< .0001		93.9 ± 15.4	107.6 ± 29.5	< .0001
HbA1c (%)	5.5 ± 0.5	6.0 ± 1.0	< .0001		5.5 ± 0.5	6.0 ± 1.0	< .0001
Smoking habit *n*, (%)	337,825 (24.1)	48,904(34.4)	< .0001		539,028 (24.3)	80,940 (34.1)	< .0001
FLI	15.8 ± 14.8	76.7 ± 11.0	< .0001		15.9 ± 15.0	76.9 ± 11.1	< .0001
Hypertriglyceridemia *n*, (%)	183,245 (13.1)	90,435 (63.6)	< .0001		302,296 (13.6)	152,122 (64.1)	< .0001
Diabetes *n*, (%)	58,152 (4.2)	28,743 (20.2)	< .0001		95,653 (4.3)	48,784 (20.6)	< .0001
Diabetes and Hypertriglyceridemia *n*, (%)	14,566 (1.0)	17,862 (12.6)	< .0001		24,735 (1.1)	30,595 (12.9)	< .0001

Among the compatible with non-NAFLD patients (*n* = 1,322,481), some patients had missing data: BMI (*n* = 1,322,314), FBG (*n* = 1,098,740), and HbA1c (*n* = 1,190,618)

Among the non-MAFLD patients (*n* = 1,400,530), some patients had missing data: FBG (*n* = 1,183,573), and HbA1c (*n* = 1,263,422)

Among the MAFLD patients (*n* = 142,158), some patients had missing data: FBG (*n* = 115,536), and HbA1c (*n* = 127,931)

*BMI* Body mass index, *FLI* fatty liver index, *LDL-C* low-density lipoprotein cholesterol, *HDL-C* high-density lipoprotein cholesterol, *TG* triglyceride, *SBP* systolic blood pressure, *DBP* diastolic blood pressure, *AST* aspartate aminotransferase, *ALT* alanine aminotransferase, *GGT*γ-glutamyl transpeptidase, *FBG* fasting blood glucose, *HbA1c* glycosylated hemoglobin

### CVD incidence rates

The numbers of cerebral infarctions, CAD events, and CVD events were 313, 2679, and 2981, respectively, among the 1,400,530 non-NAFLD patients in 4.0 years and 41, 831, and 868, respectively, among the 142,158 NAFLD patients in 4.0 years. The incidence rates of CAD were 0.87 (95% CI 0.84–0.91) and 2.70 (95% CI 2.52–2.89) per 1000 person-years in the non-NAFLD and NAFLD groups, respectively. The incidence rates of CVD were 0.97 (95% CI 0.94–1.01) and 2.82 (95% CI 2.64–3.01) per 1000 person-years in the non-NAFLD and NAFLD groups, respectively. The incidence rates of cerebral infarction were 0.10 (95% CI 0.09–0.11) per 1000 person-years and 0.13 (95% CI 0.10–0.18) per 1000 person-years in the non-NAFLD and NAFLD groups, respectively (Table 2).

**Table 2:** Incidence rate of primary outcomes in non-NAFLD and NAFLD patients

	non-NAFLD (*n* = 1,400,530)	NAFLD (*n* = 142,158)
Cerebral infarction	313	41
	0.10 (0.09–0.11)	0.13 (0.10–0.18)
Coronary artery event	2679	831
	0.87 (0.84–0.91)	2.70 (2.52–2.89)
Cardiovascular event	2981	868
	0.97 (0.94–1.01)	2.82 (2.64–3.01)

Incidence rate of primary outcomes in non-MAFLD and MAFLD patients		
	non-MAFLD (*n* = 2,215,707)	MAFLD (*n* = 237,242)
Cerebral infarction	563	81
	0.11 (0.10–0.12)	0.14 (0.12–0.18)
Coronary artery event	4675	1423
	0.90 (0.88–0.93)	2.55 (2.42–2.69)
Cardiovascular event	5217	1498
	1.01 (0.98–1.03)	2.69 (2.55–2.83)

*Top* number of events

*Bottom* incidence rate, events/1000 person-years (95% confidence interval)

The numbers of cerebral infarctions, CAD events, and CVD events were 563, 4675, and 5217, respectively, among the 2,215,707 non-MAFLD patients in 4.0 years and 81, 1423, and 1498, respectively, among the 237,242 MAFLD patients in 4.0 years. The incidence of CAD was 0.90 (95% CI 0.88–0.93) and 2.55 (95% CI 2.42–2.69) per 1000 person-years in the non-MAFLD and MAFLD groups, respectively. The incidence rates of CVD were 1.01 (95% CI 0.98–1.03) and 2.69 (95% CI 2.55–2.83) per 1000 person-years in the non-MAFLD and MAFLD groups, respectively. The incidence rates of cerebral infarction were 0.11 [95% CI 0.10–0.12] per 1000 person-years and 0.14 [95% CI 0.12–0.18] per 1000 person-years in the non-MAFLD and MAFLD groups, respectively (Table 2).

### Hazard ratio for CVD events

**Fig. 4:**
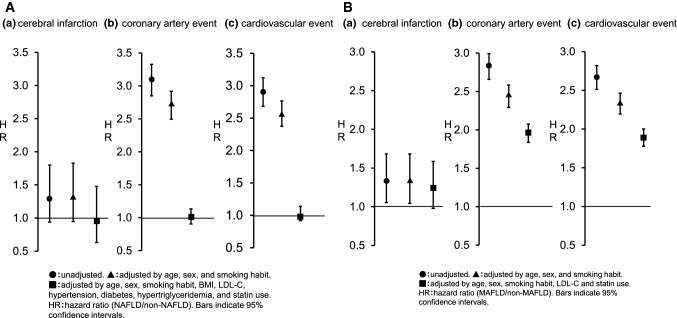
Hazard ratio for primary outcomes (**A**) NAFLD patients. ●: unadjusted. ▲: adjusted by age, sex, and smoking habit. ■: adjusted by age, sex, smoking habit, body mass index, low density lipoprotein cholesterol, hypertension, diabetes, hypertriglyceridemia, and statin use. Hazard ratios in NAFLD and non-NAFLD patients. **a** Cerebral infarction, **b** coronary artery event, and **c** cardiovascular event. **B** MAFLD patients. ●: unadjusted. ▲: adjusted by age, sex, and smoking habit. ■: adjusted by age, sex, smoking habit, low density lipoprotein cholesterol, and statin use. Hazard ratios in MAFLD and non-MAFLD patients. Bars indicate 95% confidence intervals. NAFLD: non-alcoholic fatty liver disease, MAFLD: metabolic dysfunction associated fatty liver disease. **a** Cerebral infarction, **b** coronary artery event, and **c** cardiovascular event

Without adjustments, the respective hazard ratios for cerebral infarction, CAD, and CVD were 1.30 (95% CI 0.94–1.80), 3.08 (2.85–3.33), and 2.89 (2.68–3.12) in the NAFLD group compared to the non-NAFLD group. When adjustments were made for age, sex, and smoking habit, the hazard ratios for cerebral infarction, CAD, and CVD were 1.32 (95% CI 0.95–1.83), 2.70 (2.50–2.92), and 2.56 (2.37–2.77), respectively. However, when adjustments were made for age, sex, smoking habit, BMI, LDL-C, existence of high blood pressure, existence of DM, existence of hypertriglyceridemia, and statin use, the respective hazard ratios for cerebral infarction, CAD, and CVD were 0.96 (95% CI 0.63–1.48), 1.01 (0.91–1.13), and 1.02 (0.92–1.14) in the NAFLD group compared to the non-NAFLD group (Fig. 4A).

Without adjustments, the respective hazard ratios for cerebral infarction, CAD, and CVD were 1.33 (95% CI 1.06–1.68), 2.82 (95% CI 2.66–2.99), and 2.66 (95% CI 2.51–2.82), respectively, in the MAFLD group compared to the non-MAFLD group. When adjustments were made for age, sex, and smoking habit, the hazard ratios for cerebral infarction, CAD, and CVD were 1.33 (95% CI 1.04–1.68), 2.43 (2.29–2.58), and 2.33 (2.19–2.46), respectively. Furthermore, when adjustments were made for age, sex, smoking habit, LDL-C, and statin use, the respective hazard ratios for cerebral infarction, CAD, and CVD were 1.25 (95% CI 0.98–1.59), 1.98 (1.86–2.10), and 1.89 (1.78–2.01) in the MAFLD group compared to the non-MAFLD group (Fig. 4B).

### Hazard ratios for CVD events in NAFLD patients with or without hypertriglyceridemia and/or diabetes

**Fig. 5:**
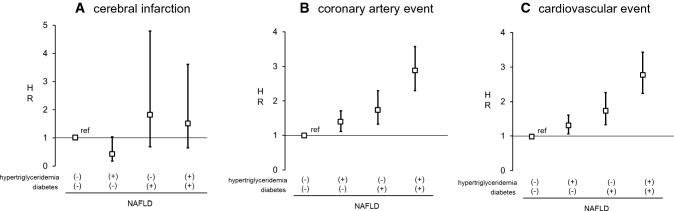
Hazard ratios of primary outcomes in NAFLD patients with or without diabetes and/or hypertriglyceridemia. **A** Cerebral infarction, **B** coronary artery event, and **C** cardiovascular event. Primary outcomes were adjusted by age, sex, smoking habit, body mass index, low density lipoprotein cholesterol hypertension, and statin use. Bars indicates 95% confidence intervals. *HR* hazard ratio, *NAFLD* non-alcoholic fatty liver disease

When adjustments were made for age, sex, smoking habit, BMI, LDL-C, existence of hypertension, and use of statin, the respective hazard rations for cerebral infarction were 0.43 (0.18–1.04), 1.82 (0.69–4.79), and 1.53 (0.65–3.61) in the presence of hypertriglyceridemia only, DM only, or both hypertriglyceridemia and DM, respectively, than in the absence of either DM or hypertriglyceridemia in the NAFLD groups (Fig. 5A).

When adjustments were made for the factors mentioned above, the respective hazard ratios for CAD were 1.38 (1.11–1.71), 1.75 (1.33–2.30), and 2.87 (2.30–3.58) in the presence of hypertriglyceridemia only, DM only, or both hypertriglyceridemia and DM, respectively, than in the absence of either DM or hypertriglyceridemia in the NAFLD groups (Fig. 5B).

Finally, the respective hazard ratios for CVD events in the presence of hypertriglyceridemia only, DM only, and both hypertriglyceridemia and DM were 1.31 (1.06–1.61), 1.74 (1.33–2.26), and 2.77 (2.24–3.43) in the NAFLD group after adjustments with similar factors (Fig. 5C).

## Discussion

This study is a cohort study conducted to verify the correlation between fatty liver defined from two different points of view: NAFLD and MAFLD, and the risk of CVD. In the present study, the incidence rates of cerebral infarction, CAD, and CVD were 0.13 (95% CI 0.10–0.18), 2.70 (2.52–2.89) and 2.82 (2.64–3.01) per 1000 person-years, respectively, in the NAFLD group. Alexander et al. reported that the incidence rates of stroke and AMI were 4.40 (4.22–4.59) and 2.07 (1.94–2.20) per 1000 person years, respectively, in the NAFLD group [14]. The difference in CVD risk between our report and previous reports might be due to regional differences [15, 16] and the proportion of elderly individuals. A representative epidemiological study in Japan (Takashima Cardio-cerebrovascular Disease Registration System and Suita study) reported that the incidence of coronary events was 1.0–2.8/1000 person-years, which is very close to the results of this study [25–27].

When adjustments were made for age, sex, and smoking habit, the hazard ratio for CVD was 2.56 in the NAFLD patients compared to the non-NAFLD patients. However, after adjustments for metabolic syndrome factors, LDL-C, and statin use, the CVD risk was almost the same (HR: 1.02) in the NAFLD and non-NAFLD groups, which is consistent with the report by Alexander et al. using a European database [14]. In this study, we also investigated the CVD risk associated with MAFLD. In 2020, an international expert panel proposed a new disease concept, "metabolic dysfunction associated fatty liver disease (MAFLD)," in which fatty liver caused by nutritional metabolic disorders can coexist with other chronic liver diseases [4]. In the MAFLD group of our study, the risk of CAD and CVD was higher than in non-MAFLD, even after adjusting by factors other than the MAFLD definition components that are associated with CVD such as age, gender, smoking status, LDL-C level, and statin use. Recently, there have been recent reports of cross-sectional observations or cohort studies showing an association between MAFLD and CVD, and in both cases, the risk ratios for CVD are higher in MAFLD populations than in NAFLD populations [12, 28, 29].

Further, we focused on DM and hypertriglyceridemia, which are important risk factors for both NAFLD, MAFLD and CVD. The role of lipogenesis in NAFLD and MAFLD development is important [18]. The accumulation of fat, mainly TG, within hepatocytes is a prerequisite for NAFLD/MAFLD development. The blockage of very-low-density lipoprotein-TG secretion, which can be caused by a dietary choline deficiency, and a reduction in fatty acid oxidation occur in the pathogenesis of nonalcoholic steatohepatitis (NASH) [30]. NAFLD, MAFLD and metabolic syndrome share many traditional, but not strictly hepatospecific, pathophysiological mechanisms. The prevalence of complications of hypertriglyceridemia only, DM only, and both hypertriglyceridemia and DM was much higher in the NAFLD and MAFLD group than in the non-NAFLD and non-MAFLD group. The risk of CVD event increased by the complication of hypertriglyceridemia and DM in the NAFLD. Additionally, combining the risk of hypertriglyceridemia and DM increased the CVD risk by about threefold in both the NAFLD groups.

Approximately 9.2% of the population (142,158/1,542,688) and 9.7% of the population (237,242/2,452,949) in this study were considered to have clinical features of NAFLD and MAFLD, respectively. Traditional cardiovascular risk factors, such as smoking habit, male sex, BMI, dyslipidemia, hypertension, and DM, were more common in the participants with NAFLD and MAFLD than in the non-NAFLD and non-MAFLD group. Previously, three cohort studies that involved 3,000–5,000 subjects, who underwent health check-ups, reported a NAFLD prevalence of 18.0–29.7% in Japan [31–33]. Additionally, Li et al. reported that the prevalence of NAFLD was 22.3% in Japan based on a meta-analysis [2]. The incidence of MAFLD has not yet been reported in detail. People who undergo annual health check-ups, included in the JMDC database, have between 20 and 60 years. Therefore, it is assumed that the incidence of NAFLD and MAFLD in this study was lower than it actually is.

The prevalence of NAFLD and NASH, and possibly of MAFLD, has increased rapidly worldwide, along with increases in the prevalence of obesity and DM [1, 2]. Currently, NAFLD/MAFLD represents one of the most important global health problems from a medical and socioeconomic standpoint. Recently, a growing body of evidence was collected to support the notion that NAFLD is both a liver specific disease and an early mediator of systemic diseases. The present retrospective longitudinal observational cohort study evaluated the incidence of CVD in 1.6—2.5 million subjects in Japan for about 4 years based on nationwide prescription records from a large administrative claims database. Real-world evidence including non-interventional studies, patient registries, claims database studies, patient surveys, and electronic health record studies, if appropriately designed, can provide valuable information concerning practice patterns and patient characteristics in actual clinical settings. The strengths of our study were the large sample size and the precise definition of CVD based on data from medical practice [34], which allowed us to accurately identify almost all patients who developed CVD during the follow-up period.

This study had several limitations. First, the JMDC Claims Database is an epidemiological receipt database that has accumulated receipts (inpatient, outpatient, dispensing) and medical examination data from several health insurance providers; therefore, the data of individuals aged under 20 years and of those aged over 65 years may be insufficient. Second, we investigated the risk of CVD by dividing the subjects into groups based on FLI, not by imaging or histopathological information. However, the use of highly objective markers such as FLI has been considered appropriate for conducting large epidemiological studies [4, 21]. FLI is thought be an important tool in epidemiological studies, particularly when assessing the incidence of NAFLD [35]. On the other hand, FLI is rarely measured in daily practice, and more than 80% of liver diseases in which other liver diseases have been ruled out are NAFLD. When classified by the presence or absence of ALT abnormalities (33 IU/L for men and 25 IU/L for women) [36], CVD was predominantly higher in the consist with NAFLD than in the non-NAFLD group (Supplementary Fig. 2 and 3, Supplementary table 5). Third, we excluded alcoholic liver disease using the ICD-10 and the results of a questionnaire to detect alcohol habits and amounts in the NAFLD study. However, the possibility of alcoholic liver disease cannot be completely excluded. Fourth, the diagnostic criteria for MAFLD are still at the stage of recommendation by the expert panel, and may change in the future. In addition, there are some items that have not been examined in this study, such as high-sensitivity CRP, HOMA-IR, and glucose tolerance test result. Finally, the JMDC Claims Database did not contain information on hepatic fibrosis. Several studies have indicated that hepatic fibrosis contributes to atherogenesis [37, 38]. Estes et al. reported that there are 3.76 million NASH patients in Japan (3% of the country’s population). Of these patients, 0.67 million people had F3/F4 fibrosis [39]. Patients with fibrosing NASH may develop CVD events and undergo an acceleration of atherosclerosis, possibly via increased hepatic production of several prothrombogenic factors, such as vascular endothelial growth factor, hypoxia-inducible factor, intracellular adhesion molecule-1, vascular adhesion molecule-1, and fetuin-A [13]. Study results may vary depending on the number of NASH cases with advanced fibrosis.

NAFLD and MAFLD are regarded as the liver component of metabolic syndrome and is reportedly associated with risk factors for metabolic syndrome. All the factors potentially involved in atherosclerotic processes are related to NAFLD and MAFLD [13, 40–43]. As discussed previously, regional differences in CVD prevalence exist worldwide [15, 16]. However, CVD is the most common cause of death in NAFLD, and possible in MAFLD patients not only in the United States and Europe, but also in Japan [44, 45]. Our study shows that patients with NAFLD and MAFLD have a higher risk of CVD.

## Supplementary Information

Below is the link to the electronic supplementary material.

Supplementary file1 (DOCX 15 KB)

Supplementary file2 (PPTX 149 KB)

Supplementary file3 (PPTX 38 KB)

Supplementary file4 (PPTX 77 KB)

Supplementary file5 (DOCX 14 KB)

Supplementary file6 (PPTX 41 KB)

Supplementary file7 (DOCX 20 KB)

Supplementary file8 (DOCX 20 KB)

Supplementary file9 (DOCX 19 KB)

## Data Availability

No additional data available.

## Abbreviations

AMI: Acute myocardial infarction
ALT: Alanine aminotransferase
AST: Aspartate transaminase
BMI: Body mass index
CVD: Cardiovascular disease
HDL-C: High-density lipoprotein cholesterol
ICD-10: International classification of diseases 10th revision
JMDC: Japan medical data center
LDL-C: Low-density lipoprotein cholesterol
MAFLD: Metabolic dysfunction associated fatty liver disease
NAFLD: Nonalcoholic fatty liver disease
NASH: Nonalcoholic steatohepatitis
TG: Triglyceride
DM: Diabetes mellitus
