# Supplementary material for: Risk of cardiovascular disease in patients with fatty liver disease as defined from the metabolic dysfunction associated fatty liver disease or nonalcoholic fatty liver disease point of view: a retrospective nationwide claims database study in Japan
Source: J Gastroenterol. 2021 Oct 3;56(11):1022–32. doi: 10.1007/s00535-021-01828-6 (PMC8531127; doi:10.1007/s00535-021-01828-6)
Supplement: Supplementary file 1 — Supplementary file1 (DOCX 15 KB) [file 535_2021_1828_MOESM1_ESM.docx]

**Supplementary Figure legends**

Supplementary Figure 1. Directed acyclic graphs for investigating causal paths for NAFLD and CVD.

Supplementary Figure 2. Flow char of consisted with compatible with NAFLD patients (Liver test abnormalities).

Supplementary Figure 3. Hazard ratios of primary outcomes in compatible with NAFLD (Liver test abnormalities) with or without diabetes and/or hypertriglyceridemia. A) Cerebral infarction, B) coronary artery event, and C) cardiovascular event. Primary outcomes were adjusted by age, sex, smoking habit, body mass index, low density lipoprotein cholesterol, hypertension, and statin use. Bars indicates 95% confidence intervals. HR: hazard ratio, NAFLD: non-alcoholic fatty liver disease.
