# Supplementary material for: Risk of cardiovascular disease in patients with fatty liver disease as defined from the metabolic dysfunction associated fatty liver disease or nonalcoholic fatty liver disease point of view: a retrospective nationwide claims database study in Japan
Source: J Gastroenterol. 2021 Oct 3;56(11):1022–32. doi: 10.1007/s00535-021-01828-6 (PMC8531127; doi:10.1007/s00535-021-01828-6)
Supplement: Supplementary file 2 — Supplementary file2 (PPTX 149 KB) [file 535_2021_1828_MOESM2_ESM.pptx]

## Slide 1
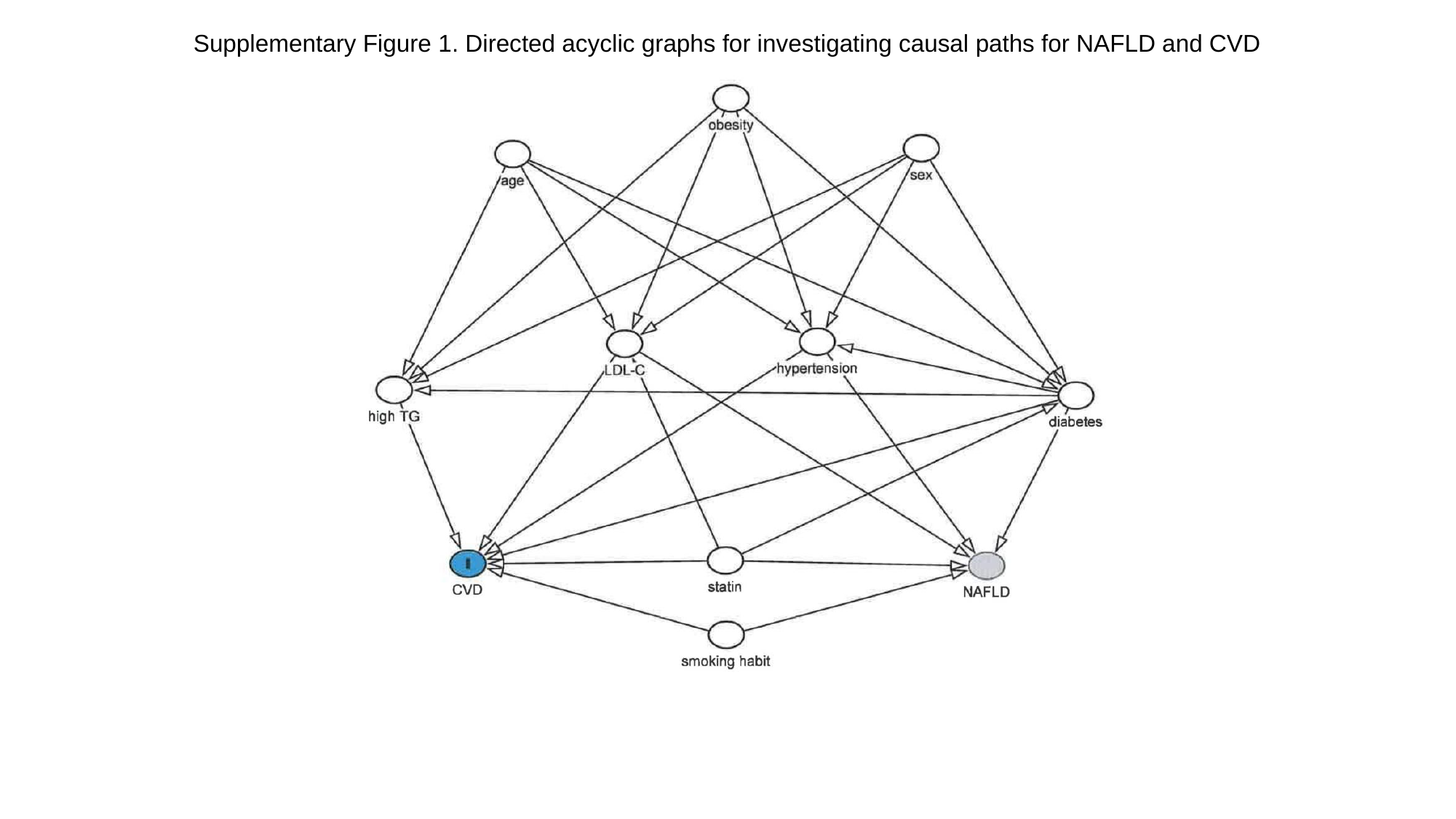

Supplementary Figure 1. Directed acyclic graphs for investigating causal paths for NAFLD and CVD
