# Supplementary material for: Risk of cardiovascular disease in patients with fatty liver disease as defined from the metabolic dysfunction associated fatty liver disease or nonalcoholic fatty liver disease point of view: a retrospective nationwide claims database study in Japan
Source: J Gastroenterol. 2021 Oct 3;56(11):1022–32. doi: 10.1007/s00535-021-01828-6 (PMC8531127; doi:10.1007/s00535-021-01828-6)
Supplement: Supplementary file 3 — Supplementary file3 (PPTX 38 KB) [file 535_2021_1828_MOESM3_ESM.pptx]

## Slide 1
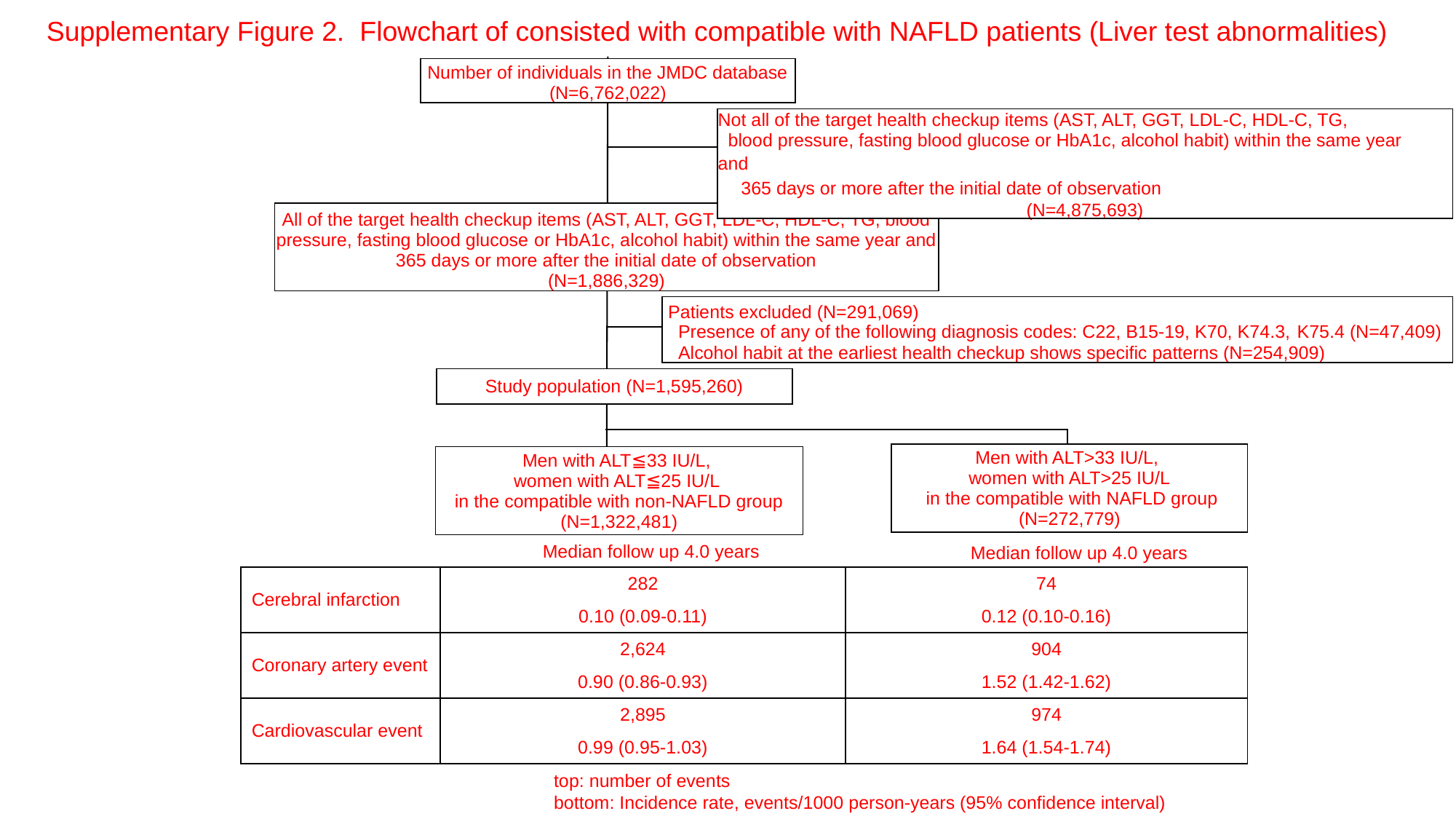

Supplementary Figure 2. Flowchart of consisted with compatible with NAFLD patients (Liver test abnormalities)
| Number of individuals in the JMDC database (N=6,762,022) |
| --- |
| Not all of the target health checkup items (AST, ALT, GGT, LDL-C, HDL-C, TG, blood pressure, fasting blood glucose or HbA1c, alcohol habit) within the same year and　 　365 days or more after the initial date of observation (N=4,875,693) |
| --- |
| All of the target health checkup items (AST, ALT, GGT, LDL-C, HDL-C, TG, blood pressure, fasting blood glucose or HbA1c, alcohol habit) within the same year and 365 days or more after the initial date of observation (N=1,886,329) |
| --- |
| Patients excluded (N=291,069) Presence of any of the following diagnosis codes: C22, B15-19, K70, K74.3, K75.4 (N=47,409) Alcohol habit at the earliest health checkup shows specific patterns (N=254,909) |
| --- |
| Study population (N=1,595,260) |
| --- |
| Men with ALT>33 IU/L, women with ALT>25 IU/L in the compatible with NAFLD group (N=272,779) |
| --- |
| Men with ALT≦33 IU/L, women with ALT≦25 IU/L in the compatible with non-NAFLD group (N=1,322,481) |
| --- |
Median follow up 4.0 years
Median follow up 4.0 years
| Cerebral infarction | 282 | 74 |
| --- | --- | --- |
| | 0.10 (0.09-0.11) | 0.12 (0.10-0.16) |
| Coronary artery event | 2,624 | 904 |
| | 0.90 (0.86-0.93) | 1.52 (1.42-1.62) |
| Cardiovascular event | 2,895 | 974 |
| | 0.99 (0.95-1.03) | 1.64 (1.54-1.74) |
top: number of events
bottom: Incidence rate, events/1000 person-years (95% confidence interval)
