# Supplementary material for: Risk of cardiovascular disease in patients with fatty liver disease as defined from the metabolic dysfunction associated fatty liver disease or nonalcoholic fatty liver disease point of view: a retrospective nationwide claims database study in Japan
Source: J Gastroenterol. 2021 Oct 3;56(11):1022–32. doi: 10.1007/s00535-021-01828-6 (PMC8531127; doi:10.1007/s00535-021-01828-6)
Supplement: Supplementary file 4 — Supplementary file4 (PPTX 77 KB) [file 535_2021_1828_MOESM4_ESM.pptx]

## Slide 1
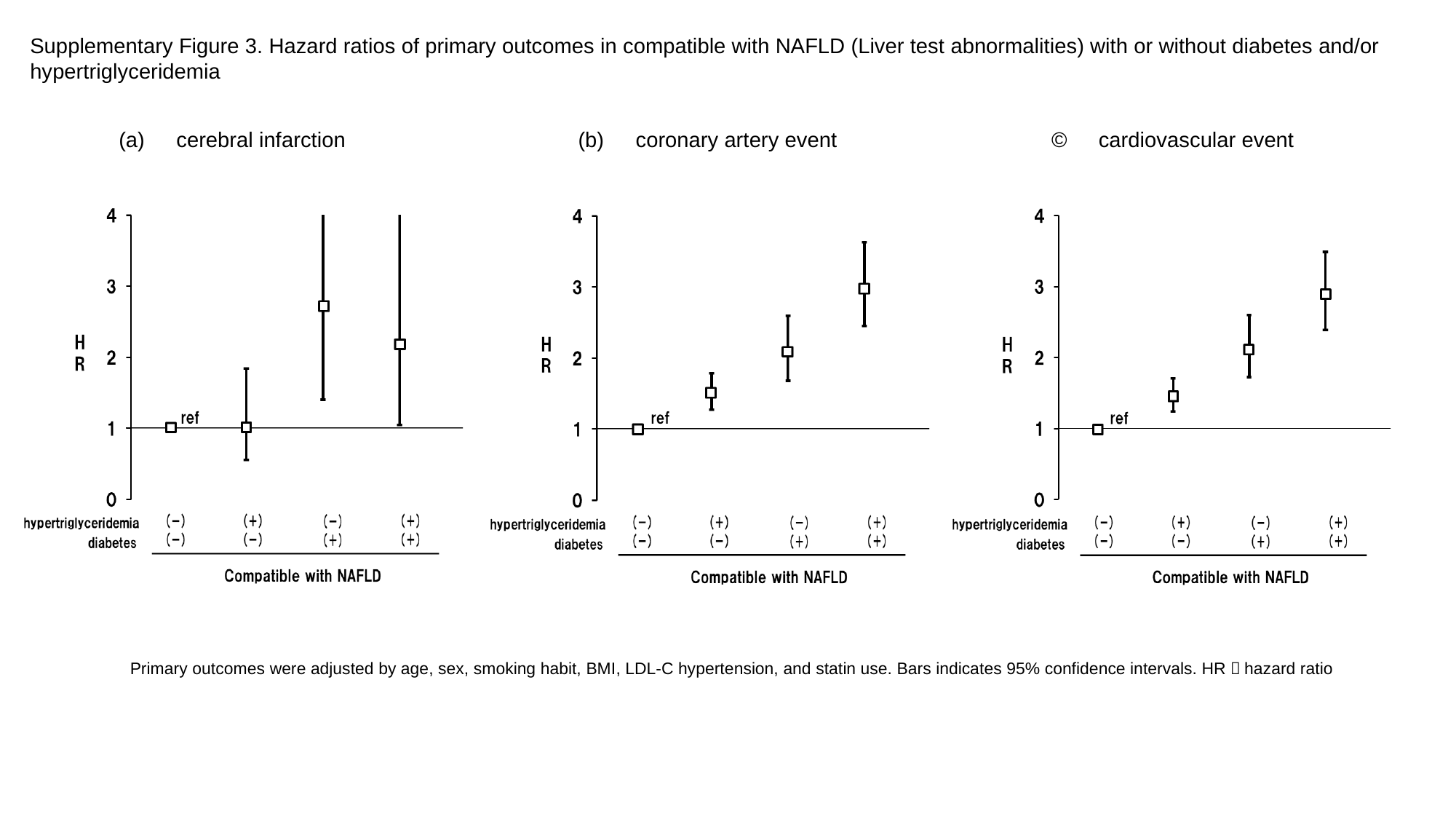

Supplementary Figure 3. Hazard ratios of primary outcomes in compatible with NAFLD (Liver test abnormalities) with or without diabetes and/or hypertriglyceridemia
©　cardiovascular event
(a)　cerebral infarction
(b)　coronary artery event
Primary outcomes were adjusted by age, sex, smoking habit, BMI, LDL-C hypertension, and statin use. Bars indicates 95% confidence intervals. HR：hazard ratio
