# Supplementary material for: Risk of cardiovascular disease in patients with fatty liver disease as defined from the metabolic dysfunction associated fatty liver disease or nonalcoholic fatty liver disease point of view: a retrospective nationwide claims database study in Japan
Source: J Gastroenterol. 2021 Oct 3;56(11):1022–32. doi: 10.1007/s00535-021-01828-6 (PMC8531127; doi:10.1007/s00535-021-01828-6)
Supplement: Supplementary file 5 — Supplementary file5 (DOCX 14 KB) [file 535_2021_1828_MOESM5_ESM.docx]

Supplementary Table.1 Questionnaire on the history of alcohol intake

| 1. How often do you consume alcoholic drinks? (sake, shochu, beer, wine, whisky, brandy, etc.)  　① everyday ② sometimes ③ rarely (do not drink) |
| --- |
| 2. How much alcoholic drink do you consume drink per day?  　① Ethanol equivalent 20g/day　② 20-40g/day ③ 60-80g/day　 ④ more than 80g/day |

We excluded participants who answered the following:

1．① and 2．③ or ④ (male)
1．① and 2．② or ③ or ④ (female)
1．② and 2．④ (male, female)
