# Supplementary material for: Risk of cardiovascular disease in patients with fatty liver disease as defined from the metabolic dysfunction associated fatty liver disease or nonalcoholic fatty liver disease point of view: a retrospective nationwide claims database study in Japan
Source: J Gastroenterol. 2021 Oct 3;56(11):1022–32. doi: 10.1007/s00535-021-01828-6 (PMC8531127; doi:10.1007/s00535-021-01828-6)
Supplement: Supplementary file 6 — Supplementary file6 (PPTX 41 KB) [file 535_2021_1828_MOESM6_ESM.pptx]

## Slide 1
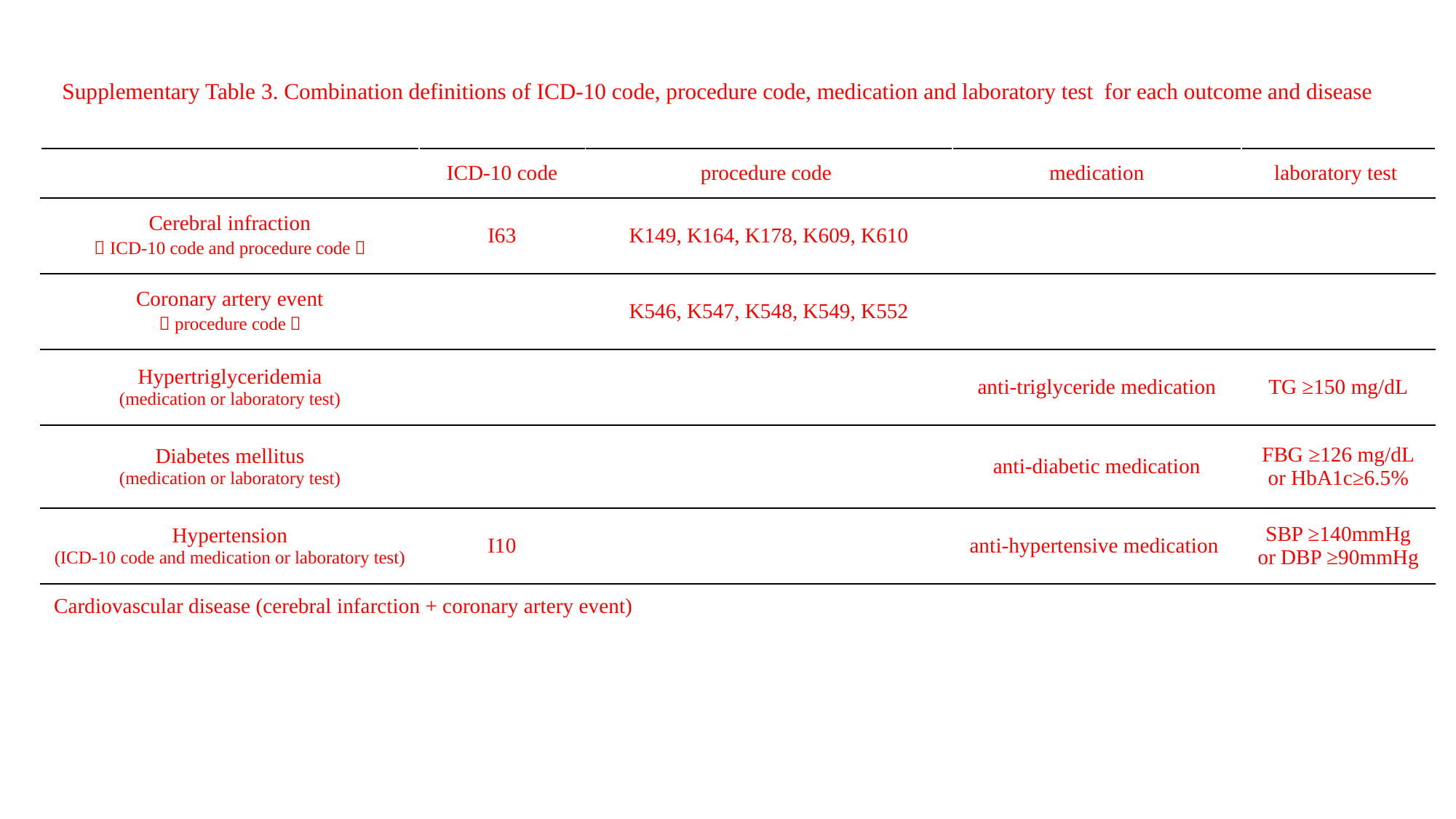

Supplementary Table 3. Combination definitions of ICD-10 code, procedure code, medication and laboratory test for each outcome and disease
| | ICD-10 code | procedure code | medication | laboratory test |
| --- | --- | --- | --- | --- |
| Cerebral infraction （ICD-10 code and procedure code） | I63 | K149, K164, K178, K609, K610 | | |
| Coronary artery event （procedure code） | | K546, K547, K548, K549, K552 | | |
| Hypertriglyceridemia (medication or laboratory test) | | | anti-triglyceride medication | TG ≥150 mg/dL |
| Diabetes mellitus (medication or laboratory test) | | | anti-diabetic medication | FBG ≥126 mg/dL or HbA1c≥6.5% |
| Hypertension (ICD-10 code and medication or laboratory test) | I10 | | anti-hypertensive medication | SBP ≥140mmHg or DBP ≥90mmHg |
Cardiovascular disease (cerebral infarction + coronary artery event)
