# Supplementary material for: Risk of cardiovascular disease in patients with fatty liver disease as defined from the metabolic dysfunction associated fatty liver disease or nonalcoholic fatty liver disease point of view: a retrospective nationwide claims database study in Japan
Source: J Gastroenterol. 2021 Oct 3;56(11):1022–32. doi: 10.1007/s00535-021-01828-6 (PMC8531127; doi:10.1007/s00535-021-01828-6)
Supplement: Supplementary file 7 — Supplementary file7 (DOCX 20 KB) [file 535_2021_1828_MOESM7_ESM.docx]

Supplementary Table 3. Medication status of study participants in the non-NAFLD and NAFLD groups

|  | non-NAFLD  (n=1,400,530) | NAFLD  (n=142,158) |
| --- | --- | --- |
| **Antidyslipidemic agents** |  |  |
| Fibrate | 12,159 (0.87) | 6,200 (4.36) |
| Nicotinic acid | 89 (0.01) | 24 (0.02) |
| EPA | 15,949 (1.14) | 3,399 (2.39) |
| Omega-3 fatty acid ethyl ester | 2,291 (0.16) | 974 (0.69) |
| Statin | 94,886 (6.78) | 23,550 (16.57) |
| Ezetimibe | 8,025 (0.57) | 3,069 (2.16) |
| PCSK9 inhibitor | 16 (0.001) | 4 (0.003) |
| **Atidiabetic agents** |  |  |
| Insulin and analogs | 7,395 (0.53) | 2,315 (1.63) |
| Hypoglycemic agent (excluding insulin) | 33,683 (2.41) | 15,739 (11.07) |
| GLP-1 analog | 633 (0.05) | 721 (0.51) |
| Biguanide | 16,548 (1.18) | 9,219 (6.49) |
| Sulfonylurea | 11,090 (0.79) | 5,300 (3.73) |
| Alpha glucosidase inhibitor | 7,790 (0.56) | 2,974 (2.09) |
| TZD | 4,306 (0.31) | 2,884 (2.03) |
| DPP-4 inhibitor | 23,877 (1.70) | 10,860 (7.64) |
| SGLT2 inhibitor | 4,239 (0.30) | 3.916 (2.75) |
| Other hypoglycemic agents | 1,990 (0.14) | 702 (0.49) |
| Aldose reductase inhibitor | 374 (0.03) | 119 (0.08) |
| **Antihypertensive agents** |  |  |
| ACEi | 8,859 (0.63) | 2,438 (1.71) |
| ARB | 68,329 (4.88) | 20,895 (14.70) |
| Ca blocker | 84,244 (6.02) | 23,978 (16.87) |
| Beta blocker | 26,465 (1.89) | 7,428 (5.23) |
| Diuretic agent | 14,075 (1.00) | 4,974 (3.50) |
| Alpha blocker | 4,665 (0.33) | 1,610 (1.13) |

ACEi: angiotensin converting enzyme inhibitor, ARB: angiotensin receptor blocker, DPP-4: **Dipeptidyl Peptidase-4,** EPA: eicosapentaenoic acid, GLP-1: Glucagon-like peptide**-1, SGLT2:** sodium glucose cotransporter, TZD: thiazolidinediones, PCSK9: proprotein convertase subtilisin/kexin type 9. Values are n (%).
