# Supplementary material for: Risk of cardiovascular disease in patients with fatty liver disease as defined from the metabolic dysfunction associated fatty liver disease or nonalcoholic fatty liver disease point of view: a retrospective nationwide claims database study in Japan
Source: J Gastroenterol. 2021 Oct 3;56(11):1022–32. doi: 10.1007/s00535-021-01828-6 (PMC8531127; doi:10.1007/s00535-021-01828-6)
Supplement: Supplementary file 8 — Supplementary file8 (DOCX 20 KB) [file 535_2021_1828_MOESM8_ESM.docx]

Supplementary Table 4. Medication status of study participants in the non-MAFLD and MAFLD groups

|  | non-MAFLD  (n=2,215,707) | MAFLD  (n=237,242) |
| --- | --- | --- |
| **Antidyslipidemic agents** |  |  |
| Fibrate | 20,831 (0.94) | 10,532 (4.44) |
| Nicotinic acid | 132 (0.01) | 49 (0.02) |
| EPA | 24,658 (1.11) | 5,675 (2.39) |
| Omega-3 fatty acid ethyl ester | 3,400 (0.15) | 1,514 (0.64) |
| Statin | 146,686 (6.62) | 37,596 (15.85) |
| Ezetimibe | 12,390 (0.56) | 5,031 (2.12) |
| PCSK9 inhibitor | 18 (0.001) | 5 (0.002) |
| **Atidiabetic agents** |  |  |
| Insulin and analogs | 12,190 (0.55) | 3,890 (1.64) |
| Hypoglycemic agent(excluding insulin) | 54,747 (2.47) | 26,755 (11.28) |
| GLP-1 analog | 994 (0.04) | 1,172 (0.49) |
| Biguanide | 26,254 (1.18) | 15,351 (6.47) |
| Sulfonylurea | 18,194 (0.82) | 9,112 (3.84) |
| Alpha glucosidase inhibitor | 12,775 (0.58) | 5,110 (2.15) |
| TZD | 7,064 (0.32) | 4,831 (2.04) |
| DPP-4 inhibitor | 38,500 (1.74) | 18,206 (7.67) |
| SGLT2 inhibitor | 6,246 (0.28) | 5.906 (2.49) |
| Other hypoglycemic agents | 3,207 (0.14) | 1,152 (0.49) |
| Aldose reductase inhibitor | 641 (0.03) | 211 (0.09) |
| **Antihypertensive agents** | | |
| ACEi | 8,493 (0.64) | 2,816 (1.03) |
| ARB | 64,535 (4.88) | 24,803 (9.09) |
| Ca blocker | 78,589 (5.94) | 29,775 (10.92) |
| Beta blocker | 25,398 (1.92) | 8,603 (3.15) |
| Diuretic agent | 13,746 (1.04) | 5,325 (1.95) |
| Alpha blocker | 4,766 (0.36) | 1,521 (0.56) |

ACEi: angiotensin converting enzyme inhibitor, ARB: angiotensin receptor blocker, DPP-4: **Dipeptidyl Peptidase-4,** EPA: eicosapentaenoic acid, GLP-1: Glucagon-like peptide**-1, SGLT2:** sodium glucose cotransporter, TZD: thiazolidinediones, PCSK9: proprotein convertase subtilisin/kexin type 9. Values are n (%).
