# Supplementary material for: Risk of cardiovascular disease in patients with fatty liver disease as defined from the metabolic dysfunction associated fatty liver disease or nonalcoholic fatty liver disease point of view: a retrospective nationwide claims database study in Japan
Source: J Gastroenterol. 2021 Oct 3;56(11):1022–32. doi: 10.1007/s00535-021-01828-6 (PMC8531127; doi:10.1007/s00535-021-01828-6)
Supplement: Supplementary file 9 — Supplementary file9 (DOCX 19 KB) [file 535_2021_1828_MOESM9_ESM.docx]

Supplementary Table 5. Clinical characteristics of study participants in the compatible with NAFLD (Liver test abnormalities)

|  | Compatible with non-NAFLD  (n=1,322,481) | Compatible with NAFLD  (n=272,779) | P value |
| --- | --- | --- | --- |
| Median (interquartile range) follow up (y) | 4.00 (2.92-5.81) | 4.00 (2.93-5.85) | <.0001 |
| Age (y) | 45.4±10.9 | 45.5±10.0 | <.0001 |
| BMI (kg/m^2^) | 22.3±3.2 | 26.0±4.3 | <.0001 |
| Male n, (%) | 798,943 (60.4) | 212,536 (77.9) | <.0001 |
| LDL-C (mg/dL) | 118.7±30.1 | 131.6±32.7 | <.0001 |
| HDL-C (mg/dL) | 64.3±16.1 | 55.0±14.9 | <.0001 |
| TG (mg/dL) | 96.0±70.0 | 152.3±114.9 | <.0001 |
| SBP (mmHg) | 118.0±15.8 | 125.3±15.8 | <.0001 |
| DBP (mmHg) | 72.7±11.5 | 78.4±11.7 | <.0001 |
| AST (U/L) | 19.5±4.7 | 33.7±16.2 | <.0001 |
| ALT (U/L) | 17.4±6.2 | 51.2±26.4 | <.0001 |
| GGT (U/L) | 27.8±23.6 | 67.1±62.2 | <.0001 |
| FBG (mg/dL) | 93.5±15.3 | 101.0±22.9 | <.0001 |
| HbA1c (%) | 5.5±0.5 | 5.8±0.8 | <.0001 |
| Smoking habit n, (%) | 324,545 (24.5) | 77,909 (28.6) | <.0001 |
| Hypertriglyceridemia n, (%) | 178,111 (13.5) | 103,090 (37.8) | <.0001 |
| Diabetes n, (%) | 54,100 (4.1) | 33,157 (12.2) | <.0001 |
| Diabetes and Hypertriglyceridemia n, (%) | 16,540 (1.3) | 16,059 (5.9) | <.0001 |

BMI: body mass index, FLI: fatty liver index , LDL-C: low density lipoprotein cholesterol, HDL-C: high density lipoprotein cholesterol, TG: triglyceride, SBP: systolic blood pressure, DBP: diastolic blood pressure, AST: aspartate aminotransferase, ALT: alanine aminotransferase, GGT:γ-glutamyl transpeptidase, FBG: fasting blood glucose, HbA1c: glycosylated hemoglobin

Among the compatible with non-NAFLD patients (n=1,322,481), some patients had missing data: BMI (n=1,322,314), FBG (n=1,098,740), and HbA1c (n=1,190,618)

Among the compatible with NAFLD patients (n=272,779), some patients had missing data: BMI (n=272,754), FBG (n=226,506), and HbA1c (n=244,009)
